# Supplementary material for: Improving routine immunization data quality using daily short message system reporting platform: An experience from Nasarawa state, Nigeria
Source: PLoS One. 2021 Aug 19;16(8):e0255563. doi: 10.1371/journal.pone.0255563 (PMC8376034; doi:10.1371/journal.pone.0255563)
Supplement: Attachment — Submitted filename: Comments_for Authors.pdf [file pone.0255563.s002.pdf]

Comments for the authors

This paper describes the launch of the RI data collection system by SMS texting and analyzes the first data points collected. These first results seem promising, despite some discrepancies among the doses opened and the effective number of children vaccinated.

I found this paper very interesting and well written. The components of the SMS texting system are clearly reported as well as the preparatory activities.
Finally, this work can help improve SMS daily tracking; it also provides practical guidelines to other countries considering implementing this type of strategy.

I have a few minor comments/details that I have listed below.

1. This discrepancy among national administrative coverage rates and effective rates is impressive. The authors comment on the possible factors that explain such difference, including erroneous data reporting, poor review, etc. I wondered if there are also intentional attempts to provide higher coverage rates, and it would be useful if the authors could add some details in the introduction.
2. In the data analysis part, I wondered which statistical analysis were conducted and if further details could be added.
3. I found it interesting that the authors recorded the highest number of reporting HFs on Tuesdays. Do the authors have some explanation about this observation?
4. Are there any plans to reduce wastage, which was particularly important for the measles case study? Also, I think that the authors suggested useful strategies for the improvement of accuracy in reporting. I wondered if there are some actual plans to understand whether the observed inconsistency is due to human error or intentional attempts at data falsification.

Specific comments:

- In the abstract, there is a missing dot at the end of the sentence: "The goal was to assess.."
- (p2) The word "post" is repeated twice in the sentence: "Community access to RI services is promoted.."
- (p2) Extra comma at the end of the sentence: "Examples are erroneous.."
- (p2) The paragraph starting with: "In this paper, we describe the launch the RI data". Shouldn't it be: "we describe the launch of the RI data?"
- (p7) The sentence starting with: "First, our SMS texting ..", there is an extra dot between "however" and "among".
- (p7) The sentence starting with: "More concerning is that ..". I think here there should be "than " instead of that (a higher number of children vaccinated *than* doses available).
- Figure 4: "OS" color bar differs from the legend
