# Supplementary material for: Improving routine immunization data quality using daily short message system reporting platform: An experience from Nasarawa state, Nigeria
Source: PLoS One. 2021 Aug 19;16(8):e0255563. doi: 10.1371/journal.pone.0255563 (PMC8376034; doi:10.1371/journal.pone.0255563)
Supplement: Attachment — Submitted filename: Response to reviewers.docx [file pone.0255563.s003.docx]

A rebuttal letter that responds to each point raised by the academic editor and reviewer(s)

Reviewer #1: The manuscript describes an assessment of data quality and cross checking data regarding to routine immunization in Nigeria through a pilot of SMS-based platform.

Although the paper is well-written (except for some typos, lower case, etc, present in the text), it presents a lack of several relevant aspects such as better overview in the initial proposed assumption. First, I would suggest a better characterization of the problem, bringing contributions of mobile health, specifically in the aspects of SMS-based communication systems. There is a huge variety of papers describing the role and impact of SMS interventions related to health. Moreover, SMS really constitutes a large spectrum of applications in health interventions, from reminders or daily checking to nudges and behavioral change. That is why would be really helpful having a better characterization of the essence of this study with other similar benchmarks. An example that reinforce this idea is the unproper way the reference 12 was placed. In that case, the object which the reference worked on it is very different of the current proposal, making difficult to support the assumption created in that part. I suggest to make a better review of it.

Response – We acknowledge the request for a better description of SMS system and the problem we are trying to address. A section has been included with two references showing the use of SMS for health interventions and a systematic review of SMS intervention. However the problem our paper is addressing is clearly specified which is using it as a reporting system from health facilities as strategy to help improve data reporting and quality in the long run

A SMS system requires a non-simple workflow involving brokers and vendors, which implies in such limitations in terms of allocated budget to carry out SMS interventions. It was not clear that SMS text cost informed ($0.01) is about the final price of vendor or the SMS landed in the system. This really matters if the paper will be used to disclose a methodology in order to be reproducible by other countries that have similar challenges. Also to understand the overall costs including the fixed-cost of an unique number for each HF it might be useful to bring more reality of financial impact of it.
Response – The comment here is well noted and we have made improvement to the section with more detail on the system infrastructure and the reason why the cost is that low. Giving the nature of the Nigerian health system the pilot was designed to lift financial burden away from the health workers however the Government of Nigeria and other partners bore the burden through the procurement of infrastructures, human resource and space for the project monitoring.

In the section "System programming development" actually discloses almost nothing about the programming development per se. If the idea is to bring technical details such workflow, programming languages, packages, modules, routines, tasks, data wrangling and data visualization, it would be recommended to bring more details about it. On the other hand, if the authors want to avoid technical terms, then the title of section should be changed.

Response – The title of this section has been changed to “Dashboard Design and Utilization”. Also the focus of the section has changed to how the dashboard is populated and the use at all levels for programme monitoring.

About the dashboard, I suggest include which were the set of metrics that were showed into it. It was only descriptive statistics or something more sophisticated was used? Any sort of early warning system was deployed? Any framework (D3.js, Shiny.r, Exploratory.io, PowerBI, Tableau, QlikView, etc) was used?

Response – This has been included in the section Dashboard Design and Utilization”. As a way to keep the dashboard user friendly, indicators monitored were basically descriptive to ensure prompt interpretation and take immediate action as necessary to address a challenge identified. The educational level of the health workers at lowest level was considered while designing the dashboard.

I think there is a lack of results in terms of metrics. I was expecting to see production metrics to prove how the system worked and scaled. For example, retention or engagement metrics are missing in the text and I'm pretty sure the authors have the raw data to calculate those.

For the analysis, why was not used any sort of simple smoothing like LOESS or GAM, or even rolling average, in order to reduce the fluctuation and bring a more consistent visualization of the series?

Response – This comment is quite helpful however its outside the scope of our objective. It will be considered in future publications on the project

A network coverage map would be useful to understand if all of HF areas were in similar covered regions, bringing more equality in the intervention.

Response – We factored the network coverage into the pilot implementation. Two districts were selected Urban (Keffi) and Rural (Keana) to ensure we can know the effect of network on the reporting. A network coverage map is not available for this study as its part of what the pilot was designed to identify. Figure 3 clear shows that network coverage was not a challenge in the reporting process.

The assumptions "For long-term sustainability independent of external support, states and LGAs will need to take ownership of the SMS reporting platform – including payment of phone credit options and maintenance of the mobile fleet." seems to simple considering the complexity of the issue. This sort of coordination sometimes is not only about funding or training, instead, if the decision makers are not involved understanding the actionable data that has been generated, this ownership will never come. I would suggest to rephrase this sentence in order to explore better the issues and show potential avenues for resolution of it.

Response – Part of the pilot was to test the coordination opportunity that reporting routine immunization data daily with SMS can bring and it was successful. Without the coordination bit, definitely the project will fail which is why we inserted this statement. Rephrasing the comment may not be necessary at this point as government at the national, state and district were involved in the processes.

This sentence "Our results can be useful for other countries’ programs that are considering SMS texting as a real-time information tool of RI activities." is not super accurate, since the presented results do not support 100% that. Also, to generate an evidence that supports this assumptions, would be necessary observe the counterfactuals as well, which was not present in this study.

Response – This is in fact true and the sentence removed accordingly.

Reviewer #2: In this work, the authors investigate a timely problem of data quality issues around routine immunization (RI) data monitoring. The paper describes the launch of the RI data collection system by SMS texting and analyzes the first data points collected.

The paper is interesting and clearly written. The SMS texting strategy has been used for the first time here to collect RI data. This paper represents an essential contribution to helping other countries that are considering implementing this kind of strategy. Besides, it highlights the significant future steps that are required to improve this type of system. Indeed, the results are promising, but some further work is needed to reach more optimal and reliable results.

I have made some minor comments for both the introduction and the methods section. Also, I found some typos in the manuscript and reported them in the “specific comments” section.
Overall, I think that this work is suitable for publication after addressing the minor comments.

Response – All comments and suggestions has been taken and effected as well.
