# Supplementary material for: Improving routine immunization data quality using daily short message system reporting platform: An experience from Nasarawa state, Nigeria
Source: PLoS One. 2021 Aug 19;16(8):e0255563. doi: 10.1371/journal.pone.0255563 (PMC8376034; doi:10.1371/journal.pone.0255563)
Supplement: Attachment — Submitted filename: Rebuttal Letter 020721.docx [file pone.0255563.s004.docx]

Response to Reviewer

Reviewer Comment - This discrepancy among national administrative coverage rates and effective rates is impressive. The authors comment on the possible factors that explain such difference, including erroneous data reporting, poor review, etc. I wondered if there are also intentional attempts to provide higher coverage rates, and it would be useful if the authors could add some details in the introduction.

Response – Our paper documented challenges with reporting from health facilities as found in previous literatures and on the field during the pilot. Adding more information may not change what is already known.

Reviewer Comment - In the data analysis part, I wondered which statistical analysis were conducted and if further details could be added.

Response - Corrected.

Reviewer Comment - I found it interesting that the authors recorded the highest number of reporting HFs on Tuesdays. Do the authors have some explanation about this observation?

Response – This is because most health facilities conduct their routine immunization sessions on Tuesday. This varies from local government to local government in Nigeria.

Reviewer Comment - Are there any plans to reduce wastage, which was particularly important for the measles case study? Also, I think that the authors suggested useful strategies for the improvement of accuracy in reporting. I wondered if there are some actual plans to understand whether the observed inconsistency is due to human error or intentional attempts at data falsification.

Response – This is a finding which is being responded to programmatically. Its out of the scope of our paper

**Specific comments:**

Reviewer Comment - In the abstract, there is a missing dot at the end of the sentence: “The goal was to assess..”

Response - Corrected.

Reviewer Comment - (p2) The word “post” is repeated twice in the sentence: “Community access to RI services is promoted..”

Response - Corrected.

Reviewer Comment - (p2) Extra comma at the end of the sentence: “Examples are erroneous.”

Response - Corrected.

Reviewer Comment - (p2) The paragraph starting with: “In this paper, we describe the launch the RI data”. Shouldn’t it be: “we describe the launch of the RI data?”

Response - Corrected.

Reviewer Comment - (p7) The sentence starting with: “First, our SMS texting ..”, there is an extra dot between “however” and “among”.

Response - Corrected.

Reviewer Comment - (p7) The sentence starting with: “More concerning is that ..”. I think here there should be “than ” instead of that (a higher number of children vaccinated *than* doses available).

Response - Corrected.

Reviewer Comment - Figure 4: “OS” color bar differs from the legend

Response - Corrected.
